# Rapid Susceptibility Testing and Microcolony Analysis of *Candida* spp. Cultured and Imaged on Porous Aluminum Oxide

**DOI:** 10.1371/journal.pone.0033818

**Published:** 2012-03-16

**Authors:** Colin J. Ingham, Sjoukje Boonstra, Suzanne Levels, Marit de Lange, Jacques F. Meis, Peter M. Schneeberger

**Affiliations:** 1 Jeroen Bosch Hospital, 's-Hertogenbosch, The Netherlands; 2 Laboratory of Microbiology, Wageningen University, Wageningen, The Netherlands; 3 MicroDish BV, Utrecht, The Netherlands; 4 Department of Medical Microbiology, Radboud University Nijmegen Medical Centre, Nijmegen, The Netherlands; 5 Department of Medical Microbiology and Infectious Diseases, Canisius-Wilhelmina Hospital, Nijmegen, The Netherlands; New Jersey Medical School, University of Medicine and Dentistry of New Jersey, United States of America

## Abstract

**Background:**

Acquired resistance to antifungal agents now supports the introduction of susceptibility testing for species-drug combinations for which this was previously thought unnecessary. For pathogenic yeasts, conventional phenotypic testing needs at least 24 h. Culture on a porous aluminum oxide (PAO) support combined with microscopy offers a route to more rapid results.

**Methods:**

Microcolonies of *Candida* species grown on PAO were stained with the fluorogenic dyes Fun-1 and Calcofluor White and then imaged by fluorescence microscopy. Images were captured by a charge-coupled device camera and processed by publicly available software. By this method, the growth of yeasts could be detected and quantified within 2 h. Microcolony imaging was then used to assess the susceptibility of the yeasts to amphotericin B, anidulafungin and caspofungin (3.5 h culture), and voriconazole and itraconazole (7 h culture).

**Significance:**

Overall, the results showed good agreement with EUCAST (86.5% agreement; n = 170) and E-test (85.9% agreement; n = 170). The closest agreement to standard tests was found when testing susceptibility to amphotericin B and echinocandins (88.2 to 91.2%) and the least good for the triazoles (79.4 to 82.4%). Furthermore, large datasets on population variation could be rapidly obtained. An analysis of microcolonies revealed subtle effects of antimycotics on resistant strains and below the MIC of sensitive strains, particularly an increase in population heterogeneity and cell density-dependent effects of triazoles. Additionally, the method could be adapted to strain identification via germ tube extension. We suggest PAO culture is a rapid and versatile method that may be usefully adapted to clinical mycology and has research applications.

## Introduction

Pathogenic fungi are a major source of human infections and are a serious risk factor for mortality. In the past, species identification has often been considered sufficient to target effective antimicrobial therapy. However, the spread of acquired resistance in both yeasts and filamentous fungi as well as persistent issues of intrinsic resistance require increased susceptibility testing [1]–[5]. Slow growth, the heterogeneous response of populations to some antimycotics, effects of inoculum density and the poor culturability of some strains are challenges for phenotypic methods. However, limitations for molecular testing include multiple mutations conferring resistance and the lack of suitable genetic markers for some drugs [6]. These considerations suggest a need for further development of rapid, culture-based susceptibility testing in fungi. For yeasts and filamentous fungi, a number of rapid phenotypic methods have been developed. These include culture in microwells containing broth with antimycotics and colorimetric redox indicators [7]–[9]. Flow cytometry has also been used to assess the action of antifungal agents with minimal incubation times [10]–[12].

In recent years there has been considerable progress in the determination and standardization of susceptibility testing for *Candida* species, including reference procedures (CLSI and EUCAST) and the study of populations of strains to determine logical breakpoints [13]. However, such procedures are quite complex and require significant expertise and resources to implement [14]. Agar diffusion methods, including commercial versions such as the E-test, are not as rigorously standardized [15], [16] but are convenient and widely used.

Microcolony detection has been used to speed up culture-based assays in fungi. For example, detection of microcolonies grown for 14 h on porous nylon membranes has been developed for viability counting of *Aspergillus fumigatus* [17]. Microcolony imaging after culture on a porous aluminum oxide support (PAO or Anopore) has been used for rapid antibiotic sensitivity testing of bacteria. Culture on PAO permits handling of intact microcolonies, including heat killing, staining with fluorogenic dyes and imaging by microscopy. Using this method, trimethoprim susceptibility has been accurately assessed in the Enterobacteriaceae within a few hours [18]. *Mycobacterium tuberculosis* can be cultured on PAO and used to determine drug susceptibility after 3 days [19]. PAO culture methods for *M. tuberculosis* have also been developed that allow monitoring during growth [20]. Given that fungi are relatively easy to image, culture on PAO may be useful for susceptibility testing of yeasts. This feasibility of this approach is supported by recent advances in low-cost imaging and analysis, i.e. microcolony quantification is becoming more accessible and automatable [21].

The aim of this study was to develop a microcolony-based method for rapid susceptibility testing of *Candida* species using a PAO support. This new approach was compared to the commonly used EUCAST and E-test methods. The objective was an assay with increased speed, reduced costs and less clinical waste.

## Results and Discussion

### Imaging and culture of *Candida* on PAO

**Figure 1:**
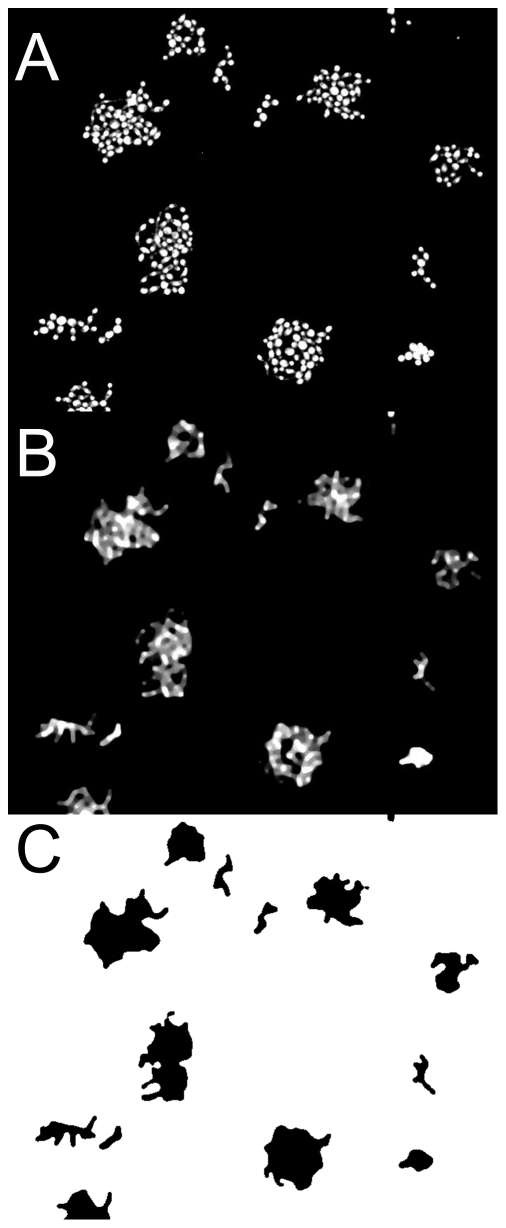
Illustration of digitization process. **A**, Raw image (100 µm across with10% area of a full picture shown) of microcolonies of *C. krusei* 870 containing up to 24 cells. **B**, Same image, now digitally processed with Median filter to merge individual cells within the same microcolony. **C**, Conversion of image from panel **B** to binary image to allow calculation of microcolony area.

**Figure 2:**
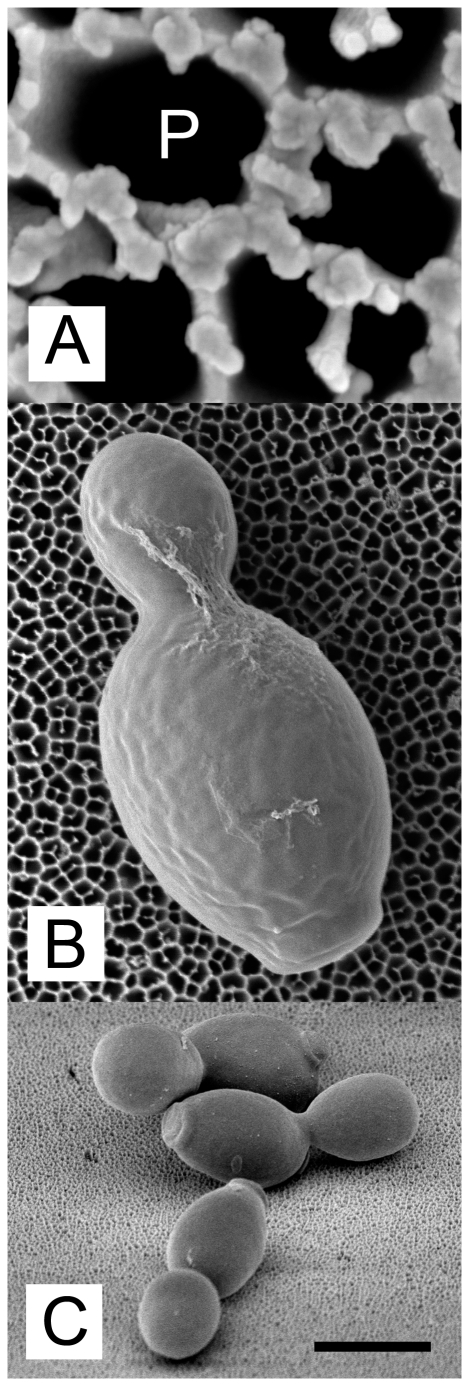
*C. albicans* strain 3003 growing on PAO and imaged by scanning electron microscopy. **A**, Structure of PAO viewed from above at high resolution. Pores (dark areas; example marked P) range from 50 to 200 nm on this face of the material. **B**, *C. albicans* growing on PAO viewed from above. **C**, *C. albicans* microcolony viewed from above, at a 45° angle. Scale bar indicates 100 nm when applied to panel **A**, 1 µm for panel **B** and 5 µm for panel **C**.

*Candida* spp. could be cultured on PAO placed on nutrient agar plates (Table 1 and Figures 1 and 2). Imaging cells stained with Fun-1/Calcofluor White was possible, using CCD camera exposures from 20 to 200 ms. Staining with a combination of Fun-1 and Calcofluor White gave an exceptionally sharp image, using a ×10 objective, with from 50 to 250 microcolonies captured per field of view. The image quality was important in automating image processing, allowing all images to be treated identically, i.e. batch processed with minimal human intervention. Stacks of up to 50 images (>5000 microcolonies) could be processed together, but 10 images (>1000 microcolonies) were sufficient for most purposes. During the time frame of these studies the microcolonies analysed within these experiments and in growth curves and for MIC testing were predominantly (>90%) monolayers. In the later stages of growth (>4 h) colonies with bilayers of cells formed. However, the microcolony area continued to increase from 4 to 6 h and was reduced in susceptible strains by triazoles; so effective MIC testing was still possible.

**Table 1:** Minimal detection times and growth on PAO.

Strain	Minimal Detection Times on PAO	Doubling Time on PAO					
	Growth	AMB	ITR	VOR	CAS	ANI	
*C. albicans* 4208	40 min	2 h	3 h	4 h	2 h	2 h	57 min
*C. glabrata* 1925	51 min	2 h	R 5 h	R 5 h	2.5 h	2 h	69 min
*C. krusei* 870	90 min	2.5 h	5 h	5.5 h	2.5 h	2 h	81 min
*C. tropicalis* 4367	87 min	2.5 h	5 h	5 h	2.5 h	2 h	91 min

	MIC Values (µg ml^−1^)				
	AMB	ITR	VOR	CAS	ANI
*C. albicans* 4208	0.25	0.016	0.016	0.5	0.008
*C. glabrata* 1925	0.25	8	16	0.5	0.016
*C. krusei* 870	0.5	0.125	0.25	0.5	0.031
*C. tropicalis* 4367	0.25	0.063	0.063	0.031	0.016

For each strain the minimal time to detect growth and the minimal time to detect drug susceptibility was calculated from triplicate time series using 8 µg AMB, VOR or ITR ml^−1^ or 4 µg CAS or ANI ml^−1^. Values are the result of triplicate determinations. The variation between such experiments was <12%. R indicates strain was resistant (so time to detection of resistance is given). Lower part of table indicates MIC values.

**Figure 3:**
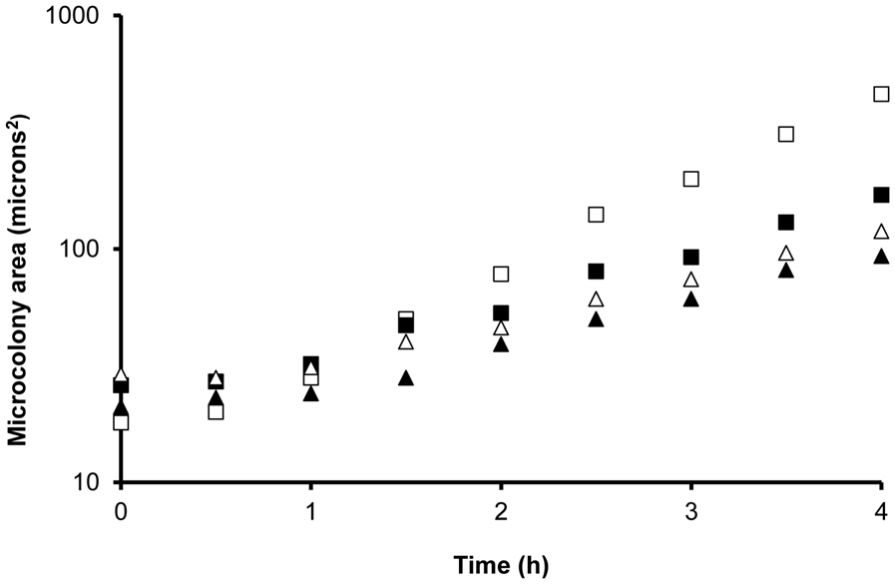
Examples of growth curves derived from microcolony areas (averaging 200 microcolonies for each data point). Open squares: *C. albicans* 4208. Closed squares: *C. glabrata* 2925. Open triangles: *C. krusei* 870. Closed triangles: *C. tropicalis* 4367. Microcolony areas at time 0 usually reflect between 1 and 2 cells.

The minimum time necessary to detect growth was calculated by imaging a series of strips of PAO incubated for different times after inoculation. A significant increase in microcolony area (P<0.05, ANOVA with Tukey *post hoc* test, N>1000 comparing later time points with T = 0) was used to assess growth. For all *Candida* species tested, significant microcolony growth on PAO was found within 91 min (Table 1). Growth curves were calculated from average microcolony areas for *C. albicans* 4208, *C. glabrata* 2925 and *C. krusei* 870 in the first 4 h of growth. *C. glabrata* 1925 had a lag time of 51 min on PAO and a doubling time of 69 min. *C. albicans* 4208 had a lag time of 40 min and doubling time of 57 min (Table 1, Figure 3). Viability testing on Sabouraud agar suggested that culturability on PAO was similar to that on agar (viable counts on PAO were 94 to 107% relative to those cells cultured directly on agar).

### Alternative staining methods

The nucleic acid stain Syto9, the metabolic dye Fun-1 and the cell wall stain Calcofluor White were all tested individually as alternatives to the combination of Fun-1/Calcofluor White. When staining microcolonies of *C. albicans* 4208 cultured for 3 h, the difference in assessment of microcolony area was minimal comparing Syto9 with Fun-1/Calcofluor White (Table 2). The variance was higher for Fun-1 in the absence of Calcofluor White. In this situation the poor definition of the cell boundary had an impact and in some cases led to single microcolonies being interpreted as clusters of microcolonies. However, all four staining methods gave MIC determinations that differed by less than two-fold (Table 2) suggesting that the dye choice is not critical.

**Table 2:** Effect of staining procedure on calculation of microcolony area and MIC determination using *C. albicans* 4208.

		PAO method with specified dye	E-test		
	Fun-1	Calcofluor	Fun+Calcofluor	Syto9	
**Average Microcolony Area** 1	113	126	131	143	
**Variance** 2	0.048	0.029	0.024	0.022	
**MIC (AMB)** 3	1	0.5	1	1	0.5
**MIC (ANI)** 3	0.128	0.256	0.128	<0.128	0.064
**MIC (CAS)** 3	0.25	0.25	<0.128	<0.128	<0.01
**MIC (ITR)** 3	0.128	0.063	<0.063	<0.063	0.023
**MIC (VOR)** 3	<0.128	0.128	0.128	<0.128	0.25

1 area in µm^2^.

2 calculated from log_10_ microcolony area.

3 MIC in µg ml^−1^.

### Surface culture of *Candida* on agar and PAO

*Candida* spp. respond to the texture and topography of their environment and are known to penetrate tissues, agar and Nuclepore membranes [22], [23]. However, cross-sections of agar plates after E-test revealed no penetration of the RPMI agar (within the area of drug action or outside it) after a 48 h assay. *C. albicans* 4208 was unable to pass through a strip of PAO, as indicated by the sterility of the Sabouraud agar surface directly beneath the PAO and by imaging. Therefore, both PAO and E-test assays are similar, in being conducted on the upper surface of the test environment.

### Susceptibility testing of *Candida* on PAO

The minimal times for the detection of susceptibility to AMB, ANI, CAS, ITR, and VOR were assessed using a concentration of each antimycotic agent above the MIC and the test panel of *Candida* species (Table 1). For AMB and CAS significant growth inhibition (ANOVA, comparing >200 microcolonies, *P*<0.05 by Tukey *post hoc* test) could be detected within 2.5 h. ANI susceptibility was detectable within 2 h. For the triazoles (ITR, VOR) the minimal time to detecting a significant effect was more variable, from 3 to 5.5 h. As a result, the standard time for PAO susceptibility testing used in subsequent work was set at 3.5 h for AMB and the echinocandins (ANI, CAS) and 7 h for the triazoles (ITR, VOR). The PAO method was tested against both EUCAST and E-test methods (Table 3). Over the entire set of five antifungal agents (170 tests per method) PAO gave good agreement with both EUCAST (86.5%) and E-test (85.9%). All three methods gave agreement in 81.2% of tests (Table 3). The best matches between PAO and both standard methods were for AMB, CAS and ANI (88.2 to 91.2% agreement). Lesser agreement was found for the triazoles ITR and VOR (79.4 to 85.3%). The datasets for VOR were recalculated using recently available population breakpoints [13] determined for *C. albicans*, *C. tropicalis* and *C. parapsilosis*. Using previously available breakpoints for these species (n = 17) the agreement with EUCAST was 14/17 and with E-test 13/17.

**Table 3:** Summary of susceptibility testing comparing PAO method with EUCAST and E-test methods.

Antifungal	PAO vs EUCAST	PAO vs E-TEST	EUCAST vs E-TEST	ALL TESTS
**AMB**	91.2	88.2	91.2	82.4
**CAS**	88.2	88.2	88.2	82.4
**ITR**	82.4	79.4	85.3	79.4
**VOR**	79.4	82.4	82.4	76.5
**ANI**	91.2	91.2	88.2	85.3
**AVERAGE**	**86.5**	**85.9**	**86.5**	**81.2**

Results of testing the panel of 34 strains noted in the Methods against AMB, CAS, ITR, VOR, and ANI. The percentage of exact matches was scored (S [sensitive] vs S, R [resistant] vs R, and where relevant I [intermediate] vs I) for pairwise comparisons between different tests and perfect agreement between all three tests.

An additional triazole, FLU, was tested for 10 strains of *C. albicans* scored as susceptible by the standard methods. The PAO method scored 6/10 of these clinical isolates as susceptible after 3.5 h and 9/10 after 7 h. Four FLU resistant strains of *C. krusei* were also tested. Of these four strains, three were scored as resistant after 3.5 h and all four after 7 h. Taken together, these data suggest that the method may be extended to testing against FLU using a similar methodology to that for ITR and VOR.

Data analysis in these testing used batch processing of each data point (different combinations of drug/strains) as separate stacks of images. This took 20–30 min per MIC determination. However, further automation of the MIC determination is possible via simple scripting methods, which suggests that data analysis will not be a major factor in the time to result in the future.

### Germ tube outgrowth on PAO

Culture of 10 clinical isolates of *C. albicans* on PAO on sheep's blood agar allowed germ tube outgrowth to be detected with 90 min incubation, a similar time period to that needed by a human observer. However, culture on PAO followed by Fun-1/Calcofluor White staining and imaging allowed a degree of automation of the evaluation. The criteria used for evaluation were aspect ratio, microcolony area and frequency. Whilst germ tube extension produced an aspect ratio that was on average longer than budding cells there were confounding factors that required additional criteria. Firstly, some strains produced a subpopulation of elongated cells on Sabouraud medium that also passed these criteria. However, such cells were rare (<5% population) and false positives were excluded by the requirement that >20% of cells had an appropriate aspect ratio. These criteria scored all 10 strains as producing germ tubes on PAO on blood agar and none on PAO on Sabouraud agar. We were unable to reproduce these results by imaging unstained cells (data not shown): the high image quality of Fun-1/Calcofluor White staining was critical to obtaining good image analysis.

### Changes in microcolony heterogeneity on PAO

**Figure 4:**
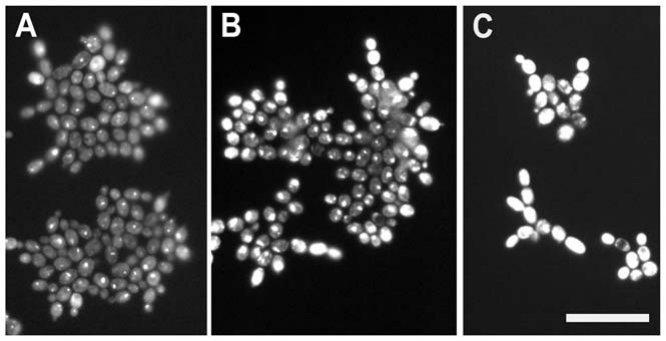
The effect of VOR on Fun-1 staining patterns of *C. tropicalis* cells within microcolonies. **A**, *C. tropicalis* 2526 (VOR sensitive, MIC 0.0625 µg ml^−1^) grown for 7 h on PAO (no-drug control). **B**, Microcolonies of the same strain cultured on 0.0625 µg VOR ml^−1^ with an increase in Fun-1 staining (particularly for cells on the periphery). **C**, As panel **B**, showing the enhancement of Fun-1 staining of peripheral cells by 0.125 µg VOR ml^−1^. Pictures were taken with identical exposure times (45 ms). Scale bar indicates 20 µm for all images.

**Figure 5:**
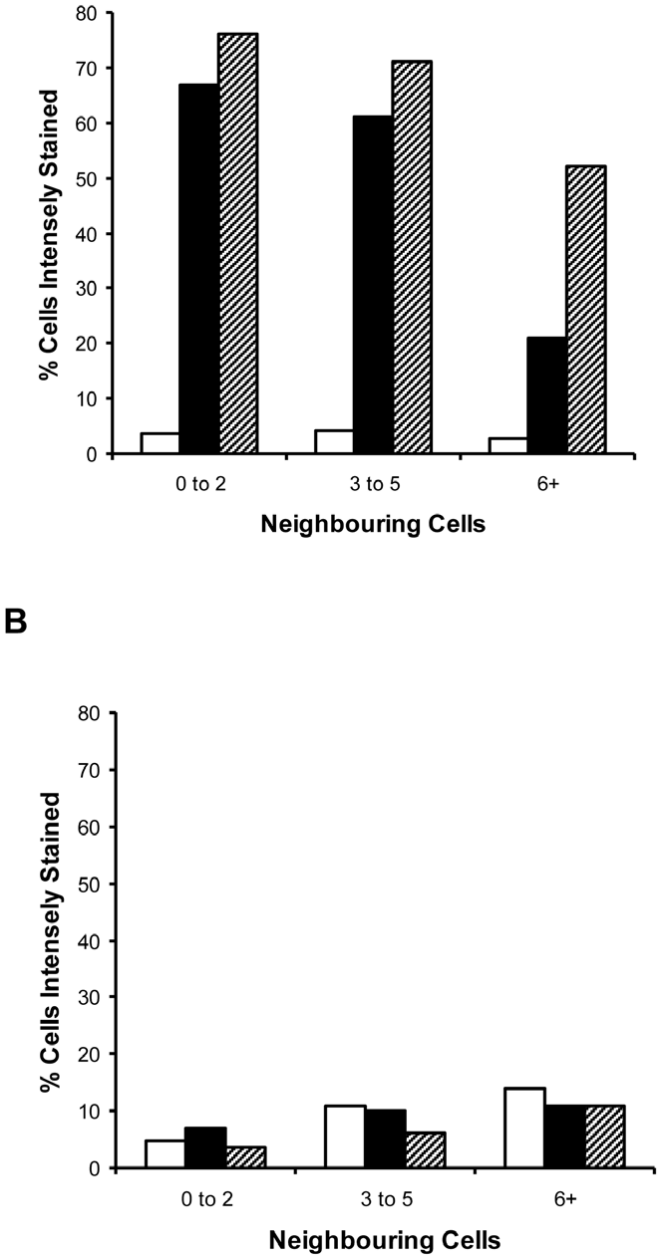
Effect of the number of flanking cells and the effect of sub-MIC concentrations of antimycotics on Fun-1 staining intensity. Positional effects of Fun-1 staining were analyzed, scoring cells staining intensely throughout (likely to be dead or stressed) and relating this to the number of adjacent cells. **A**, *C. tropicalis* 2526 (VOR sensitive, MIC 0.0625 µg ml^−1^) grown for 7 h on PAO. White bars, no-drug control. Black bars, culture with 0.0625 µg VOR ml^−1^. Hatched bars, culture on 0.125 µg VOR ml^−1^. **B**, *C. tropicalis* 8404 (VOR resistant, MIC 8 µg ml^−1^), VOR concentrations and shading as panel **A**.

When scoring susceptibility it was noticed that Fun-1 staining was enhanced in sensitive strains growing in the presence of triazoles at concentrations close to the MIC. Cells were often strongly and uniformly stained when at the periphery of microcolonies with >10 cells or when part of smaller microcolonies. This trend was not seen with VOR- or ITR-resistant strains. Furthermore, when cells were viewed by fluorescence microscopy, the variation in staining intensity appeared unusually large even within sibling cells forming individual microcolonies and therefore separated by at most three rounds of division. For example, the mean microcolony area was identical for strain 2526 of *C. tropicalis* cultured with 0 and 0.0625 µg VOR ml^−1^ but the mean staining intensity and heterogeneity of staining within individual microcolonies was elevated in the presence of VOR. Therefore, VOR and ITR appeared to be having a significant effect on cells at levels below those required to limit growth. The center of the microcolonies showed pinpoint staining whilst the periphery showed strong but even staining (Figure 4). A spatial analysis was made, scoring each of 300 cells (>80 microcolonies) for staining pattern. In sensitive strains, sub-lethal VOR stress elevated the number of intensely staining cells, particularly cells with few immediate neighbors – i.e. small microcolonies or cells on the periphery of large microcolonies (Figure 5). Typically, cells in the interior of microcolonies with 20+ cells had from 6 to 8 immediate neighbors. As with the qualitative analysis, this trend was weak or absent in a VOR-resistant (MIC 8 µg ml^−1^) strain using the same concentration of VOR. The data presented in Figures 4 and 5 are derived from *C. tropicalis* but the same staining pattern was detectable in other species including *C. albicans* (data not shown) and for ITR and FLU as well as VOR.

Fun-1 is a halogenated unsymmetrical cyanine dye that fluoresces when complexed with proteins and does so more intensely when bound to nucleic acids. Sequestration of metabolic products of Fun-1 within CIVS is the most common result for healthy yeasts [24], [25]. Staining of CIVS is punctate and intense. A more uniform and intense fluorescence is associated with cell stress or death. Enhancement of mean Fun-1 staining [10], [11] and increased heterogeneity (C. Pina Vaz, personal communication) has also been observed by FACS analysis of triazole treated *Candida* spp. The increased staining intensity was attributed to the blocking of efflux pumps that export the drug [10], [11]. Microcolony imaging allowed this observation to be refined: sublethal concentrations of triazoles were apparently more stressful to cells on the periphery of a microcolony than to those in the interior.

In non-pathogenic yeasts, phenotypic variation has been shown to be a valuable survival strategy in fluctuating environments [26]. It is possible that this is also the case in *Candida* spp. For example, in generating individual cells with an above-average ability to export a toxic compound. However, the positional effects suggest that this is not a random increase in phenotypic variation, but that the interior of even a small microcolony (<20 cells) may be a safer place to be during limited triazole exposure. Given that the limited number of cells and nutrients and triazoles supplied from beneath the cells directly through the high-porosity PAO, we suggest this phenomenon is unlikely to be due to poor access to nutrients or reduced drug concentrations. From viewing microcolonies of different proximity to each other (e.g. Figure 4B) it appeared that the apparent protection is short-ranged (<10 microns) but does not necessarily require cells to be touching. These data suggests that microcolony analysis reveals the behaviour of subpopulations that are not amenable to other methods.

Testing *Candida* spp. for triazoles required a longer culture time for sensitivity to be reliably detected than tests for AMB, ANI or CAS, for which 3.5 h culture was used as standard. Incubation for 7 h was required for a consistent MIC determination with ITR and VOR (Table 1). This was also the case for FLU. The triazoles are fungistatic for *Candida* spp., with trailing effects caused by continued growth of a proportion of sensitive cells. For all the antimycotics the time taken to result was rapid compared to E-test: tests for AMB and the echinocandins could be completed within 5 h whilst the triazoles could be assayed within a normal working day or overnight. We suggest that another advantage of this test is its versatility. To date we have looked at a wide range of bacteria [18]–[20] and fungi and a chemically diverse range of antimicrobials. Furthermore, other dyes such as Syto9 were effective in accurately determining MICs. We suggest that this toolbox can readily be adapted to new antimicrobials or new organisms. Finally, as the method is based around cell imaging it may be able to cope with agents such as echinocandins in filamentous fungal for which the primary assay is a change in morphology (minimal effective concentration or MEC).

Microcolony assays can be used to look at heterogeneity within populations. Such analysis reveals a degree of variance within genetically homogeneous populations as well as spatial effects. Fun-1 is a particularly interesting dye for this purpose as it shows very different staining patterns depending on the stage of conversion and sequestration [25]. However, as the susceptibility assay is primarily dependent on calculation of microcolony area such heterogeneity of conversion does not have a major impact as long as the contrast between stained cells and edge of the colony is sufficient to allow accurate measurements, which was the case in our experiments (Table 2).

The large datasets obtained from this analysis (up to 1000 microcolonies) permit the detection of subtle trends, such as the density-dependent effects [27] of antimycotics that are less obvious than bulk inhibition of growth or killing. In this work we observed shifts in the distribution of populations in response to levels of antimycotics. Whilst there was very little change in the mean microcolony area, the variance of the microcolony area increased. Increase in microbial heterogeneity is often a response to stress. Such information may prove useful in understanding both the mechanism of resistance and phenotypic heterogeneity, particularly with reference to effective dosage of antimycotics.

### Conclusions

Clinical isolates of a range of *Candida* spp. grew as rapidly on PAO as on agar, and after a few hours the resultant microcolonies could be stained and quantified. Microcolony imaging on PAO allowed the creation of rapid culture-based assays and the MIC testing on *Candida* on surfaces may be more relevant to how these yeasts exist in the human body than planktonic growth, as used in broth dilution methods. Poor nutrient environments with oxygen limitation and the potential for biofilm development can be created on PAO, which may allow further development to increase the relevance of MIC testing *in vitro* to clinical outcomes. A microcolony-based susceptibility assay was developed, giving accurate results with 3.5 to 7 h culture, compared with 24 to 48 h by E-test. Agreement between the PAO method and the two standard approaches was good (c. 86% in both cases). Triazoles were somewhat harder to test, in terms both of requiring a longer incubation and of slightly lower accuracy compared to the results for the echinocandins and AMB (Table 3) but could also give good results. The clinical gains from increasing the speed of conventional methods (24–48 h) requires investigation – but we note *Candida* can be life threatening on an immediate basis (e.g. sepsis) so there are obvious applications for this method. Analysis is via simple computer scripts and plug-ins for publicly available, free software (ImageJ) and further automation is possible. This method has the advantages of speed and versatility; currently the cost per test is less than 15 dollars, though integration of multiple tests on a single PAO strip has the potential to decrease this. Further standardization is required for the introduction of our method into routine clinical practice. Developments in low-cost LED imaging may support readout systems that are cheaper and improve standardization, allowing use of the technique in a routine setting. Significant morphology changes such as germ tube extension can also be scored from growing microcolonies, suggesting that this method of strain identification is feasible on PAO. Microcolony analysis gives significant data on the state of individual cells within populations, variation that conventional tests do not reveal. We noted that sensitive cells within the interior of microcolonies appeared less affected by triazoles than cells at the periphery. We suggest that the PAO microcolony imaging method for susceptibility can also be adapted to other fungi. To develop this test further the testing of multiple antimicrobials on a single strip of PAO is desirable and imaging without staining would improve the workflow.

## Methods

### Strains and culture

*Candida* spp. were obtained as clinical isolates from the JB Hospital, Den Bosch, The Netherlands, with reference strains from the Mycology Reference Laboratory (Bristol, UK). A panel of 34 strains (11 strains of *C. albicans*, 2 *C. dubliniensis*, 9 *C. glabrata*, 6 *C. krusei*, 2 *C. parapsilosis* and 4 *C. tropicalis*) was used. Clinical isolates were identified by Auxacolor (Bio-Rad Laboratories, Veenendaal, The Netherlands) and germ tube extension in serum. Additional strains were three amphotericin B-resistant isolates derived from UV mutagenesis of strain JBZ111 and repeated selection on Sabouraud agar containing 4 µg amphotericin B ml^−1^. Routine culture of all strains was on Sabouraud dextrose agar at 37°C.

### Antifungal drugs

Amphotericin B (AMB; Bristol Myers Squibb, Woerden, The Netherlands), voriconazole (VOR; Pfizer Central Research, United Kingdom), itraconazole (ITR; Janssen Cilag, The Netherlands), caspofungin (CAS; Merck Sharp & Dohme BV, The Netherlands), fluconazole (FLU; Sigma Chemicals, The Netherlands) and anidulafungin (ANI; Pfizer Central Research) were used in this study. The drugs were obtained as reagent-grade powders and were preserved according to the manufacturer's instructions.

### MIC determinations by standard methods

The minimal inhibitory concentrations (MICs) or minimum effective concentrations were determined using a broth microdilution method, according to the reference procedure of the Antifungal Susceptibility Testing Subcommittee of EUCAST (http://www.eucast.org/). The E-test was implemented using RPMI-1640 plates and according to the manufacturer's instructions and recommended breakpoints (E-test, BioMerieux, Boxtel, The Netherlands) with incubation for 48 h.

### Preparation, culture and imaging on PAO

PAO strips (8×36 mm, 60 µm thick, pore size 200 nm; manufactured by Whatman, Maidstone, UK) for the culture of fungi were sterilized by dry heat; 170°C for 2 h [19], [28]. Sterile strips, and strips divided into up to 6 culture areas, were stored in tubes and deployed onto nutrient agars by gently tapping the tube. Dilutions of clinical isolates were made in sterile phosphate-buffered saline; these were inoculated onto PAO placed on agar plates from 2,000 to 20,000 colony-forming units (CFU) mm^−2^. Agar plates used were RPMI-1640 medium (all components Sigma, Heerhugowaard, The Netherlands), Sabouraud dextrose (Oxoid, Basingstoke, UK) or sheep's blood agar (Oxoid) with incubation at 37°C. Staining of fungi was with Calcofluor White/FUN-1 and Syto9 (Invitrogen, Breda, The Netherlands). Dyes were diluted into molten 1% (w/v) low-melting-point agarose (10 µM Syto 9, 20 µM Fun-1, 0.05% (w/v) Calcofluor White as appropriate). Aliquots (1 ml) of the relevant dye mix were poured evenly on a microscope slide and allowed to solidify. The PAO was transferred to the slide for staining and imaged on the same slide after 20 min. Image capture used an Olympus BX-41 microscope equipped with a ×10 Fluorotar objective lens and an 8 bit Kappa black-and-white CCD camera [29]. Growth curves were calculated from microcolony areas (>400 for each data point).

### Agar and PAO penetration assays

Vertical cross-sections of agar (1 mm thick, 2 cm across, 0.6 mm high), taken from Sabouraud plates after E-tests, were made with a sterile razor blade. The region sectioned included the zone of clearing created by the antimycotics and the flanking regions of vigorous growth. Such cross-sections were placed on slides covered with solidified low-melting-point agar containing 20 µM FUN-1 [25], then incubated for 40 min in a humidified environment and imaged for evidence of penetration of the agar by *Candida* spp. Assays for the penetration of PAO were done by inoculating sterile PAO on Sabouraud or sheep's blood agar with 10^3^ to 10^5^ CFU of *C albicans* JBZ111 and incubating for 1 to 6 h at 37°C. After removal of the PAO, penetration of the agar beneath was assessed by further incubation of the agar plate (16 h) and direct imaging by microscopy. Imaging used Olympus BX-41 fluorescence microscope equipped with U-MWIBA filters (excitation spectrum of 460 to 490 nm, dichroic mirror splitting at 505 nm, and an emission spectrum of 515 to 550 nm, used for Syto9 and Fun-1 dyes), U-M41007 (530 to 560 nm excitation, 565 nm splitting, and 575 to 645 nm emission, used for PI and HI dyes), and U-M41008 (590 to 650 nm emission, 660 nm splitting, and 665 to 735 nm excitation) (Olympus, Japan). An Olympus V-MNV2 filter with excitation of <420 nm was used for visualizing Calcofluor White staining.

### Image processing and statistical analysis

Greyscale 8-bit TIFF images were processed as individual images or in stacks using ImageJ software (http://rsbweb.nih.gov/ij/). Processing used a median filter (radius 4 pixels) followed by a binary threshold adjustment (Fig. 1). The ImageJ “analyze particle” function was then used to calculate microcolony areas, excluding particles too small to be cells and those only partially within the field of view. Statistical analysis used ANOVA with a Tukey *post hoc* test calculated by the Vassar statistics server (http://faculty.vassar.edu/lowry/VassarStats.html). For large-scale analysis (>5 images), stacks of up to 50 images were created using ImageJ and then batch processed. The intrapopulation variance in microcolony area was calculated after a log_10_ transformation of the microcolony area [30] calculated from at least 1000 microcolonies. The variance in Fun-1 staining was derived from non-saturated, 8-bit TIFF images, calculating the average signal intensity per cell using ImageJ cell-counter plug-ins. Heterogeneity analysis was also performed within microcolonies, looking at positional effects on the staining pattern. In this case each cell was scored for (a) the number of cells it had as immediate neighbours and (b) the type of staining pattern. Staining type was scored as intense, with even, strong staining throughout the cell; punctate, with pinpoint staining of cylindrical intravacuolar structures (CIVS); or weak (cell wall stain brighter than interior stain) as described by Millard et al. [25].

### MIC testing on PAO

MIC testing was done on RPMI-1640 plates containing defined concentrations of antimycotics with up to four PAO strips per plate, up to six samples per strip. MIC values were calculated from the decrease in average microcolony areas from at least five images (>1000 microcolonies) for each data point. The number of microcolonies to be analyzed was chosen on the basis of detecting an average 1.3-fold increase in microcolony area (P<0.05, Student's *t*-test). The method was similar to that previously described for Enterobacteriaceae [19]. Fun-1/Calcofluor White staining was used unless stated otherwise.

#### Germ tube outgrowth on PAO

Inoculation for germ-tube formation was as for other culture experiments on PAO but using sheep's blood agar. Staining and data capture were as above. Image processing used specific criteria to identify germ-tube-producing cells: an aspect ratio of >3.2 and longest dimension of 3 to 8 µm. A strain was scored as positive if at least 20% of the objects analyzed passed this test.

### Scanning electron microscopy (SEM)

Cells were cultured and fixed with glutaraldehyde (2.5% v/v) on PAO, then treated with osmium tetroxide (1% w/v), dehydrated and subjected to critical point drying as previously described [18]. Imaging was done with an FEI Magellan electron microscope (FEI Company, Eindhoven, The Netherlands) after tungsten sputtering.
